# Piperine inhibits ABCA1 degradation and promotes cholesterol efflux from THP‐1‐derived macrophages

**DOI:** 10.1002/mnfr.201500960

**Published:** 2016-12-23

**Authors:** Limei Wang, Veronika Palme, Susanne Rotter, Nicole Schilcher, Malsor Cukaj, Dongdong Wang, Angela Ladurner, Elke H. Heiss, Herbert Stangl, Verena M. Dirsch, Atanas G. Atanasov

**Affiliations:** ^1^ Department of PharmacognosyUniversity of ViennaViennaAustria; ^2^ Institute of Medical ChemistryCenter for Pathobiochemistry and GeneticsMedical University of ViennaViennaAustria; ^3^ Institute of Genetics and Animal Breeding of the Polish Academy of SciencesJastrzebiecPoland

**Keywords:** ABCA1, Calpain, Cholesterol efflux, Macrophages, Piperine

## Abstract

**Scope:**

Increased macrophage cholesterol efflux (ChE) is considered to have anti‐atherosclerotic effect counteracting cardiovascular disease. The principle pungent ingredient of the fruits of *Piper nigrum*, piperine, is identified in this study as a ChE inducer in THP‐1‐derived macrophages, and mechanisms underlying this effect are explored.

**Methods and results:**

Without affecting cell viability, piperine concentration‐dependently enhances ChE in THP‐1‐derived macrophages from 25 to 100 μM. The expression level of the key cholesterol transporter protein ATP‐binding cassette transporter A1 (ABCA1) is significantly upregulated by piperine, as revealed by western blot analyses. However, two other ChE‐mediating transporter proteins, ATP‐binding cassette transporter G1 (ABCG1) and scavenger receptor class B member 1 (SR‐B1), remain unaffected. Piperine exerts no influence on ABCA1 mRNA levels, but significantly inhibits the degradation of ABCA1, as evident by an increased half‐life of the protein in the presence of cycloheximide. Furthermore, it is found that piperine likely interferes with the calpain‐mediated ABCA1 degradation pathway and exhibits significant inhibition of calpain activity.

**Conclusion:**

Our findings suggest that piperine promotes ChE in THP‐1‐derived macrophages by upregulation of ABCA1, which might be mediated by inhibition of calpain activity. This novel bioactivity makes the dietary constituent piperine a good candidate to be further explored for therapeutic or preventive applications in the context of atherosclerosis.

**Abbreviations**
ABCA1: ATP‐binding cassette transporter A1
ABCG1: ATP‐binding cassette transporter G1
CAPNS1: calpain small subunit 1
ChE: cholesterol efflux
CHX: cycloheximide
FBS: fetal bovine serum
FC: free cholesterol
LXR: liver X receptor
PEST: proline, glutamic acid, serine, and threonine rich
PMA: phorbol 12‐myristate 13‐acetate
PPARγ: peroxisome proliferator activated receptor gamma
qRT‐PCR: quantitative real time PCR
RCT: reverse cholesterol transport
SR‐B1: scavenger receptor class B member 1

## 1 Introduction

Atherosclerosis‐related cardiovascular disease is the main contributor to mortality and morbidity worldwide. Accumulation of cholesterol in macrophages is an important process in the pathogenesis of atherosclerosis. The reverse cholesterol transport (RCT) is reported to prevent the development of atherosclerosis by transportation of peripheral cholesterol to the liver for excretion into bile and ultimately feces 1, 2, 3. Cholesterol efflux (ChE), the initial step of RCT, comprises the export of cholesterol from macrophages or other cell types. Thereby increased ChE is regarded to have an antiatherosclerotic effect due to the elimination of excessive intracellular cholesterol. ChE involves the transport of accumulated intracellular cholesterol to extracellular lipid poor apolipoprotein (apo) A1 or HDL 1, 2, 4.

Four possible ChE pathways have been described: aqueous diffusion, scavenger receptor class B member 1 (SR‐B1), ATP‐binding cassette transporter A1 (ABCA1), and ATP‐binding cassette transporter G1 (ABCG1) mediated ChE 5. Three of these pathways are mediated by transporter proteins (ABCA1, ABCG1, and SR‐B1). Free cholesterol (FC) transported through ABCA1 is the predominant pathway for ChE mediated by the ABC family of transmembrane transporters, which contributes to approximately two thirds of the ChE in macrophages 5. Further studies have shown that the intracellular FC effluxed through ABCA1 predominantly binds to apo A1, while FC effluxed through ABCG1 preferentially binds to HDL to enter the next steps of the RCT 2, 5, 6.

Considering the importance of ABCA1 protein in mediating ChE, the transcriptional as well as posttranslational regulation of ABCA1 has been widely studied in recent years. The transcription of ABCA1 is enhanced by activation of ligand‐dependent nuclear receptors such as peroxisome proliferator activated receptor gamma (PPARγ) and liver X receptor (LXR) 7, 8. Posttranslational regulation of ABCA1 protein levels might be achieved by modulation of its degradation rate. Three prevailing pathways are implied in ABCA1 degradation in different cell types, particularly the proteasome‐, lysosome‐ and calpain‐mediated protein degradation pathways 9, 10, 11, 12. Among these pathways, the calpain‐mediated ABCA1 degradation is reported to be of importance in THP‐1 macrophages 13, due to sequence rich in proline, glutamic acid, serine, and threonine (PEST sequence) in the ABCA1 protein that can be recognized by calpain, leading to the degradation of ABCA1 11.

Natural products have been a continuous source of therapeutic agents historically, and still represent an important pool for the discovery of new drug leads 14. Piperine is the principle pungent constituent of the fruits of *Piper nigrum* L., which is one of the most widely used spices worldwide 15, 16. Piperine is abundant in green pepper, black pepper, and white pepper, which correspond to the different harvesting stages of the fruits of *P. nigrum* 17. To be noted, piperine is not only present in the seeds, but also abundant in the other vegetative parts of *P. nigrum* 18. Besides the prominent food usage, pepper fruits also have documented applications in traditional Chinese medicine for the treatment of vomiting, abdominal pain, diarrhea, and anorexia due to cold in the stomach, and epilepsy with much phlegm 19. Recent research also shows that piperine exhibits beneficial cardiovascular effects in vivo. In rats fed on a high‐fat diet enriched with cholesterol and other lipids, elevated plasma levels of HDL and apo A1, and decreased plasma levels of total cholesterol, triglyceride, and LDL cholesterol (LDL cholesterol) were observed in animals supplied with piperine‐supplemented food 20, 21, 22, 23. Another study found that rats fed with high carbohydrate, high‐fat diet supplemented with piperine displayed normalized blood pressure and improved aortic reactivity 24.

In this study, we show that piperine enhances ChE in THP‐1‐derived macrophages and further explore its mechanism of action, shedding new light on potential cardiovascular effects of the traditionally used pepper spice.

## 2 Materials and methods

### 2.1 Chemicals and reagents

Piperine (#P49007), digitonin (#D141), cycloheximide (CHX) (#C7698), lactacystin (#L6785), calpeptin (#C8999), chloroquine diphosphate salt (#C6628), phorbol 12‐myristate 13‐acetate (PMA) (#P1585), apolipoprotein A1 (apo A1) (#73366), and water‐soluble unesterified cholesterol (#C4951) were purchased from Sigma‐Aldrich (Vienna, Austria), and pioglitazone (#M35102242) was obtained from Molekula (Munich, Germany). [^3^H]‐cholesterol (#NET139001MC; 1 mCi, 37 MBq) was provided by Perkin Elmer Life Sciences (Vienna). Human plasma was kindly supplied by Prof. Herbert Stangl, Medical University of Vienna. The tested compounds were dissolved in DMSO, aliquoted, and stored at −20°C until use. An equal amount of DMSO was always tested under each condition in all experiments to assure that the solvent vehicle does not influence the results itself.

Primary antibodies against ABCA1 (#NB400‐105), ABCG1 (#NB400‐132), SR‐B1 (#NB400‐104), and calpain small subunit 1 (CAPNS1; #NBP1‐90329) were obtained from Novus Biologicals (Vienna). The anti‐calpain 1 (#sc‐7531) and ‐calpain 2 (#sc‐373967) antibodies were from Santa Cruz Biotechnology (Dallas, USA). The anti‐total actin antibody (#8691002) was acquired from MP biologicals (Illkirch, France) 25, 26. HRP‐linked anti‐rabbit IgG secondary antibody (#7074S) was purchased from New England Biolabs (UK), and horseradish peroxidase conjugated goat anti‐mouse secondary antibody (#12‐349) from Upstate (Millipore, Vienna). All antibodies were used in a dilution of 1:500. Silencer® Pre‐Designed siRNA (#AM16704) targeting CAPNS1 was obtained from Thermo Fisher Scientific.

### 2.2 Cell culture and differentiation

Human monocytic THP‐1 cells were obtained from the ATCC and maintained in RPMI‐1640 medium (Lonza, Basel, Switzerland) with 10% fetal bovine serum (FBS; Gibco, Lofer, Austria), 2 mM l‐glutamine, 100 U/mL penicillin, and 100 μg/mL streptomycin in a humidified atmosphere of 5% CO_2_ at 37°C. THP‐1 monocytes were cultured in T175 flasks (Sarstedt, Austria). For the experiments described below, THP‐1 monocytes were differentiated into macrophages by culturing the cells at a density of 0.2 × 10^6^ cells/mL in the presence of 200 nM PMA for 72 h. Further details of the particular cell culture conditions in each experiment are stated in the following methods’ description.

### 2.3 Cell viability evaluation

Cell viability was evaluated with resazurin conversion 27 as previously described 28. THP‐1 monocytes were seeded in 96‐well plates (100 μL per well of a suspension containing 0.2 × 10^6^ cells/mL) and differentiated into macrophages with 200 nM PMA for 72 h. After incubation with the indicated concentrations of piperine or digitonin (cytotoxic natural product used as a positive control) in FBS free medium supplemented with 0.1% BSA and 10 μg/mL unesterified cholesterol for 24 h, the cell were washed once with prewarmed PBS and further incubated in freshly prepared resazurin (10 μg/mL) dissolved in PBS for another 4 h. The relative cell viability was quantified from the increased fluorescent signal by the conversion product resorufin by measuring the fluorescence at 580 nm emission/535 nm excitation with a Tecan GENiosPro plate reader (Männedorf, Switzerland).

### 2.4 ChE assay

The ChE assay 27 was established based on previously published studies 7, 8. Briefly, 0.2 × 10^6^ THP‐1 monocytes per well were seeded and differentiated into macrophages in 24‐well plates in the presence of 200 nM PMA for 72 h. Cells were supplemented in FBS‐free RPMI‐1640 medium with 0.1% BSA and 10 μg/mL unesterified cholesterol and treated with the indicated compounds (piperine from 5 to 100 μM and pioglitazone at 10 μM) together with 0.3 μCi [^3^H]‐cholesterol for 24 h to acquire dynamic equilibration. After incubation, cells were washed twice with warmed PBS (without calcium and magnesium) and divided into two groups. One group was treated with either 10 μg/mL apo A1 or 1% human plasma (to reconstitute the physiological situation in which exported cholesterol is transferred to apo A1 or plasma HDL), and the other group was treated with just the indicated compounds in FBS‐free medium. Cells were incubated for 6 h to allow the ChE to take place. Then, both radioactivity inside and outside of the cells was measured by liquid scintillation counting. Apo A1‐ and 1% human plasma‐mediated ChE were calculated as follows 8:

Apo A1 mediated  ChE %= extracellular  cpm  apo A1 total  cpm  apo A1− extracellular  cpm  no  apo A1 total  cpm  no  apo A1×100

Human  plasma  mediated  ChE %=× extracellular  cpm  plasma  total  cpm  plasma − extracellular  cpm  no  plasma  total  cpm  no  plasma ×100

### 2.5 Protein extraction and western blotting

THP‐1 macrophages (0.8 × 10^6^/well) were seeded in 6‐well plates and first treated with the indicated concentrations of piperine and pioglitazone for 24 h in FBS‐free RPMI‐1640 medium supplemented with 0.1% BSA and 10 μg/mL unesterified cholesterol. After incubation, cells were lysed with NP40 buffer (150 mM NaCl; 50 mM HEPES (pH 7.4); 1% NP40; 1% protease inhibitor Complete (Roche); 1% PMSF; 0.5% Na_3_VO_4_; 0.5% NaF) for 30 min at 4°C. The lysed cells were collected and centrifuged at 16 060 g for 20 min to collect the supernatant. Protein concentration was assessed with Bradford assay using Roti^®^‐Quant from Carl Roth (#K015.1, Karlsruhe, Germany). Total protein (20 μg per sample) was loaded and separated via SDS‐PAGE. Expression levels of specific proteins were analyzed using antibodies against the indicated proteins and visualized with ECL reagent and a LAS‐3000 luminescent image analyzer (Fujifilm) with AIDA image analyzer 4.06 software (Raytest). A representative whole‐blot picture resulting from the detection of ABCA1 in the presence and absence of piperine is presented in the Supporting Information Fig. 1.

### 2.6 RNA extraction and quantitative real‐time PCR (qRT‐PCR)

Total RNA was extracted from differentiated THP‐1 macrophages using peqGOLD Total RNA Kit (PeqLab, Linz, Austria) according to the manufacturer's instructions. The quantitation of RNA was performed with NanoDrop 2000C (peqlab, Thermo Scientific) and A_260/280_ close to 2.0 was considered to indicate sufficient RNA quality. cDNA was synthesized with 1 μg total RNA based on the protocol from the High Capacity cDNA Reverse Transcription Kit (Applied Biosystems) with RNase Inhibitor (Applied Biosystems). qRT‐PCR was conducted using the LightCycler^®^ 480 SYBR Green I Master kit (Roche) with 40 ng cDNA for each sample. The LightCycler 480 system from Roche was used for detection of the amplification cycles. Primers used for the qRT‐PCR were specific for ABCA1 (HS_ABCA1_1_SG QuantiTect Primer assay, Cat. no.: #QT00064869, QIAGEN) and 18S (Hs_RRN18S_1_SG QuantiTect Primer assay, Cat. no.: #QT00199367, Qiagen). Relative quantification of ABCA1 gene expression was performed with the Δ*C*_T_ method, using 18S as endogenous control.

### 2.7 Calpain activity determination

THP‐1 monocytes were seeded in 6‐well plates (4 mL/well of a cell suspension with density of 0.2 × 10^6^ cells/mL), and differentiated into macrophages for 72 h with 200 nM PMA. The differentiated macrophages were treated with 50 μM piperine for 24 h and then the cells were collected for calpain activity detection according to the standard protocol from the Calpain Activity Assay Kit (fluorometric) (#ab65308, Cambridge, UK). Calpain activity detection was performed with 80 μg protein per reaction. Z‐LLY‐FMK (10 μM; directly added to lysates from DMSO‐treated cells) was used as a positive control. Black 96‐well plates with clear bottom were used for measurement with a Tecan Infinite 200^®^ Pro plate reader (Vienna) at a wavelength Ex/Em = 400/505 nm.

### 2.8 Statistical analysis

All experiments in this study were performed at least in three independent experiments (*n* = 3) with newly differentiated cells. One‐way analysis of variance (ANOVA) and two‐way ANOVA were used for statistical analysis of the acquired data from different conditions using GraphPad Prism software version 4.03 (GraphPad Software Inc., La Jolla, CA, USA). Results are presented as mean ± SD. The differences were considered significant when *p* < *0.05*. Further details for each experiment are provided in the respective figure legends.

## 3 Results

### 3.1 Piperine promotes ChE from THP‐1 macrophages

**Figure 1:**
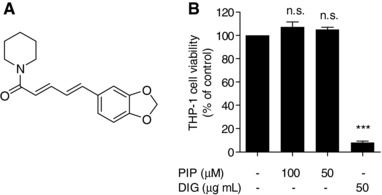
Chemical structure of piperine (A) and its effect on the viability of THP‐1 macrophages (B). Differentiated THP‐1 macrophages were incubated with solvent vehicle control (DMSO), 50 μg/mL digitonin (DIG; cytotoxic natural product) as a positive control, or the indicated concentrations of piperine (PIP). After 24 h, cell viability was determined by the resazurin conversion method.  All values are mean ± SD (*n* = 4) versus solvent vehicle control, ***p < 0.001; n.s., no significance (one‐way ANOVA/Bonferroni).

**Figure 2:**
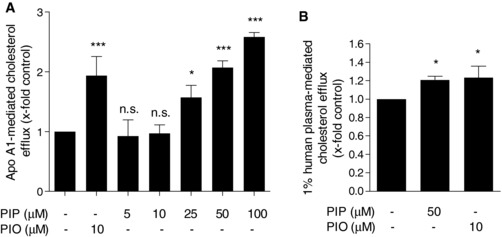
Effect of piperine on ChE in THP‐1 macrophages. Differentiated THP‐1 macrophages were loaded with [^3^H]‐cholesterol together with solvent vehicle (DMSO), piperine (PIP, 5–100 μM), or the PPARγ agonist pioglitazone (PIO, 10 μM) as a positive control as indicated for 24 h. On the next day, cells were washed twice with PBS and incubated again with the same compounds in the presence or absence of 10 μg/mL apo A1 (A) or 1% human plasma (B) dissolved in serum‐free medium, for 6 h. Extracellular as well as intracellular radioactivity were quantified by scintillation counting. All values are mean ± SD (*n* = 3) versus solvent vehicle control, *p < 0.05; ***p < 0.001; n.s., no significance (one‐way ANOVA/Bonferroni).

First we surveyed whether piperine (Fig. 1A), applied at concentrations that did not affect cell viability (Fig. 1B), can increase ChE in differentiated THP‐1 macrophages. As evident in Fig. 2A, piperine enhances apo A1‐mediated ChE concentration dependently (5–100 μM). Pioglitazone, a PPARγ agonist, applied at 10 μM was used as positive control since it is a well‐known macrophage ChE inducer 29. Furthermore, piperine also increased ChE mediated by human plasma (Fig. 2B), which is the acceptor with highest relevance to the ChE process happening in vivo.

To determine the optimal concentration for subsequent experiments we specified THP‐1 cell viability at the most effective concentrations of piperine (50 and 100 μM) in comparison to the cytotoxic natural product digitonin [(50 μg/mL); used as positive control]. Figure 1B highlights that neither 50 nor 100 μM piperine affects cell viability whereas 50 μg/mL digitonin caused around 90% decrease in cell viability. Accordingly, 50 μM piperine was used in subsequent experiments, since at this concentration it displayed a highly significant ChE‐promoting effect without affecting cell viability.

### 3.2 Piperine increases ABCA1 protein level

**Figure 3:**
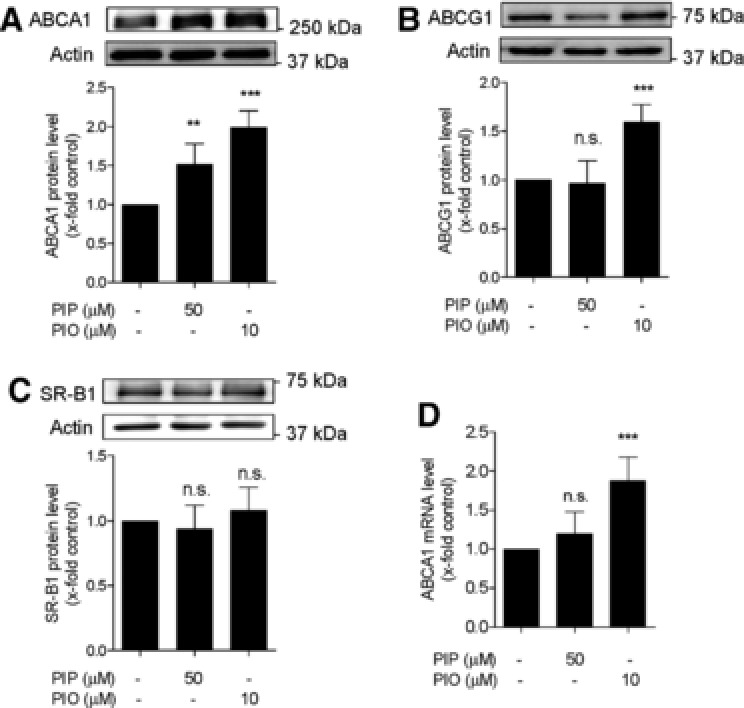
Expression of cholesterol transporter proteins in the absence and presence of piperine. (A–C) Differentiated THP‐1‐derived macrophages were treated with solvent vehicle control (DMSO; indicated as not treated with piperine or pioglitazone), piperine (PIP, 50 μM), or the PPARγ agonist pioglitazone (PIO, 10 μM) as positive control. After 24 h incubation, cells were lysed and 20 μg protein was resolved via SDS‐PAGE. Immunodetection was performed with antibodies against the indicated proteins, ABCA1 (A), ABCG1 (B), and SR‐B1 (C), and the protein bands were visualized by chemiluminescence detection. All values are mean ± SD (*n* = 4) versus solvent vehicle control, ***p < 0.001; n.s., no significance (one‐way ANOVA/Bonferroni). (D) Differentiated THP‐1 macrophages were incubated with 50 μM piperine (PIP) and 10 μM pioglitazone (PIO) for 24 h. Total RNA was extracted and ABCA1 mRNA expression levels were quantified by qRT‐PCR. Data are mean ± S.D. (*n* = 5) versus solvent vehicle control (DMSO), **p < 0.01; ***p < 0.01; n.s., no significance (one‐way ANOVA/Bonferroni.

ABCA1, ABCG1, and SR‐B1 are the most important transporter proteins for ChE in THP‐1 macrophages. To investigate how piperine increases ChE, we first tested the expression levels of these three transporter proteins. Piperine significantly induced apo A1‐mediated ChE in THP‐1 macrophages (Fig. 2A) and it is known that ABCA1 exports intracellular cholesterol most efficiently to apo A1 5. Indeed, Fig. 3A shows that ABCA1 protein levels are significantly upregulated upon piperine (50 μM) treatment consolidating a significant role for this transporter in apo A1‐mediated ChE. To determine whether piperine acts selective on ABCA1, we also examined the expression levels of ABCG1 and SR‐B1 proteins in the presence of piperine. However, neither ABCG1 nor SR‐B1 was affected upon piperine treatment (Fig. 3B and C). Pioglitazone, known to augment ABCA1 and ABCG1 protein levels in macrophages 29, was used as positive control.

To determine whether the upregulated ABCA1 protein expression correlates with increased mRNA level qRT‐PCR experiments were performed. As shown in Fig. 3D, piperine at 50 μM is not significantly altering ABCA1 mRNA level after 24 h treatment, while pioglitazone (10 μM) significantly augments ABCA1 transcription as reported previously 29. Time‐course experiments tracking ABCA1 mRNA levels also revealed no significant effect of piperine at any of the investigated time points (0, 3, 6, 15, 24 h; data not shown).

### 3.3 Piperine inhibits ABCA1 degradation

**Figure 4:**
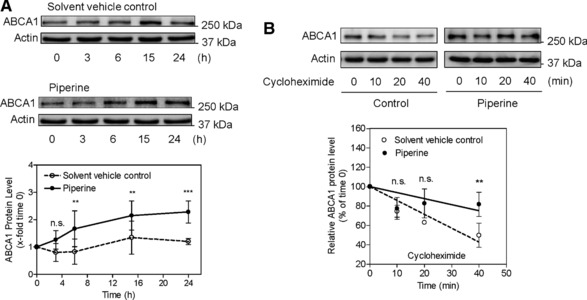
Effect of piperine on ABCA1 protein‐dependent expression and the degradation rate of ABCA1 protein. (A) Differentiated THP‐1 macrophages were incubated up to 24 h with (black circles) or without (solvent vehicle control; white circles) piperine at 50 μM. Cells were lysed at different time points (0, 3, 6, 15, 24 h) and proteins were resolved on SDS‐PAGE. Immunodetection was performed with the indicated antibodies. All data are mean ± SD (*n* = 3) versus solvent vehicle control (DMSO), **p < 0.01; ***p < 0.001; n.s., no significance (two‐way ANOVA/Bonferroni). (B) Differentiated THP‐1 macrophages were incubated for 24 h with (black circle) or without (solvent vehicle control; white circle) piperine (50 μM) and lysed after addition of CHX (140 μM) at different time points (0, 10, 20, 40 min). Western blot analysis shows the decline of ABCA1 protein levels in the CHX‐treated cells in the presence and absence of piperine. All data are mean ± SD (*n* = 3) versus solvent vehicle control (DMSO), **p < 0.01; n.s., no significance (two‐way ANOVA/Bonferroni).

Increased protein levels in the absence of increased mRNA levels might be attributed to reduced protein degradation, since the total ABCA1 protein amounts reflect a balance between cellular synthesis and degradation 30. Therefore, we evaluated the degradation rate of ABCA1 in the absence and presence of piperine. To specify a suitable incubation time, time‐course experiments in respect to ABCA1 protein expression in the presence and absence of piperine were performed (Fig. 4A) 30. Upon treatment with piperine (50 μM), ABCA1 protein levels were increased in a time‐dependent manner, compared to the solvent vehicle control (DMSO), with the first significant effect displayed 5 h after treatment, and the most abundant protein levels acquired after 24 h. Thus, THP‐1 macrophages were first treated with piperine (50 μM) for 24 h to allow protein synthesis and were then treated with 140 μM CHX, an inhibitor of de novo protein synthesis, in order to block further protein generation 13, and then lysed at different time points (0, 10, 20, 40 min). ABCA1 protein abundance at the different time points was determined by western blotting analysis (Fig. 4B). Under these conditions (in the presence of CHX), changes in ABCA1 protein levels reflect an altered rate of degradation. Notably, piperine is slowing down the degradation rate of ABCA1, when compared with the solvent vehicle (Fig. 4B). A significantly different ABCA1 level in the piperine‐treated conditions was evident 40 min after the application of CHX with around 80% protein remaining compared with about 50% remaining under solvent vehicle treatment. Thus, piperine inhibits ABCA1 protein degradation, which is likely to contribute to the ChE‐promoting effect of piperine.

### 3.4 Piperine might interfere with calpain‐mediated ABCA1 degradation

**Figure 5:**
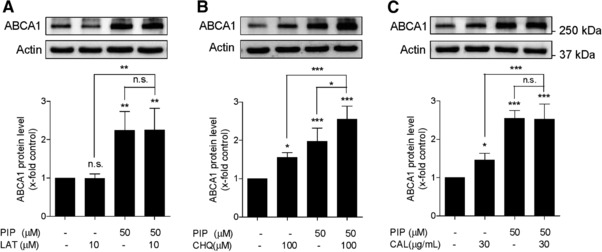
Effects of piperine and specific protein degradation pathway inhibitors on the ABCA1 protein levels. Differentiated THP‐1 macrophages were pretreated with or without piperine (PIP) at 50 μM for 24 h and incubated for another 3 h with (A) the proteasome inhibitor lactacystin (LAT) at 10 μM, (B) the lysosomal inhibitor chloroquine (CHQ) at 100 μM, or (C) the calpain inhibitor calpeptin (CAL) at 30 μg/mL. Cells were lysed and proteins were resolved on SDS‐PAGE. Depicted representative western blots show ABCA1 protein levels under the indicated treatment. All data are mean ± SD (*n* = 4) versus solvent vehicle control (DMSO), *p < 0.05; **p < 0.01; ***p < 0.001; n.s., no significance (one‐way ANOVA/Bonferroni).

**Figure 6:**
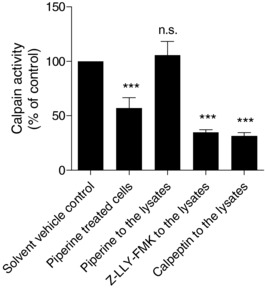
Effect of piperine on calpain activity. Differentiated THP‐1 macrophages were incubated for 24 h with or without (solvent vehicle control) piperine at 50 μM. Samples from cells were extracted and calpain activity was assessed as described in Section 2. An irreversible inhibitor of calpain (Z‐LLY‐FMK; 10 μM) and calpeptin (30 μg/mL) was used as positive control. All data are mean ± SD (*n* = 4) versus solvent vehicle control (DMSO), ***p < 0.001; n.s., no significance (one‐way ANOVA/Bonferroni).

Based on previous reports, three prevailing pathways are involved in ABCA1 degradation, the proteasome‐, lysosome‐, and calpain‐mediated protein degradation pathways 9, 10, 11, 12. In order to study the mechanism by which piperine inhibits ABCA1 degradation, a putative role of these three pathways was further investigated by the application of specific pathway inhibitors in the presence and absence of piperine (Fig. 5). As shown in Fig. 5A, under the used experimental conditions the protein level of ABCA1 remained unaffected in the presence or absence of lactacystin, a proteasome inhibitor abolishing the trypsin‐like, chymotrypsin‐like, and peptidylglutamyl‐peptide peptidase activities of the proteasome complex 31. In the presence of 50 μM piperine, the abundance of ABCA1 protein remained also unchanged with or without lactacystin. At the same time, the application of calpeptin (a calpain inhibitor) and chloroquine (a lysosome inhibitor) increased ABCA1 protein level, as did also piperine (Fig. 5B and C). Piperine had an additive effect on ABCA1 protein level upon cotreatment with chloroquine, indicating that its mechanism of action is likely different than that of the lysosomal inhibitor chloroquine (Fig. 5B). However, there was no difference observed with or without calpeptin in the presence of piperine, suggesting that piperine might interfere with the calpain degradation pathway, possibly mimicking the effect of calpeptine (Fig. 5C). This result is consistent with former findings 9, 11, 32 demonstrating that calpain plays an important role in ABCA1 degradation, especially in THP‐1 macrophages 13. To test the hypothesis for a calpain‐inhibitory action of piperine, calpain activity was directly assessed in THP‐1 lysates. Indeed, application of piperine (50 μM; 24 h) resulted in a significant suppression of calpain activity (Fig. 6). Importantly, direct application of piperine (50 μM) to cell lysates fails to inhibit calpain enzyme activity, indicating that the mechanism of action of the compound requires long‐term cell exposure and is not based on direct calpain inhibition (Fig. 6). Furthermore, piperine also fails to reduce protein levels of calpain 1 and calpain 2 (Supporting Information Fig. 2), the archetypical and ubiquitously expressed members of the calpain family, which are also reported to have a functional significance in macrophages 33, 34, 35, 36. Moreover, a siRNA knockdown of the CAPNS1, a heterodimer subunit essential for the function of both calpain 1 and calpain 2, did not affect the ABCA1‐stabilizing effect of piperine (Supporting Information Fig. 3).

## 4 Discussion

The present study shows for the first time that piperine increases ChE from human THP‐1‐derived macrophages and inhibits the degradation of the ChE transporter ABCA1, likely by reducing its calpain‐mediated degradation.

Given the broad use of black pepper as spice all over the world, piperine, its most distinct constituent has also already gained significant research attention in the context of cardiovascular disease and a number of in vivo studies suggest that it has antiatherosclerotic activity 21, 22, 23, 24, 37. While macrophage ChE enhancement is one possible way for a small molecule to exert an antiatherosclerotic effect, the ChE‐inducing potential of piperine has not been studied so far.

Piperine is commonly found in a variety of daily used foods and condiments including black pepper, white pepper, or green pepper, and is generally considered as safe in its common dietary applications. In our cell model, we also did not see cytotoxicity for piperine in the used concentration range (up to 100 μM). The half maximal effective concentration (EC_50_) of the piperine‐mediated ChE induction is 37.4 μM, which is a high concentration that is unlikely to be reached in the bloodstream upon dietary piperine exposure. Nevertheless, it is important to note that piperine has a good oral bioavailability, and interestingly is even broadly used as enhancer, coadministrated with other drugs in order to increase their bioavailability 38, 39, 40. The concentration of piperine in black pepper spiced soup (1 g black pepper powder/200 mL soup) was determined to be around 771 μM (44 mg piperine per 200 mL soup), and upon its consumption from healthy volunteers the maximally reached serum levels were in the low micromolar range 41. While considering the significance of our findings for potential future therapeutic applications, it should be noted that possible effects of chronic intake of lower piperine concentrations cannot be directly extrapolated from our work and would need to be addressed with in vivo studies. In the context of cellular cholesterol utilization it is also reported that piperine concentration dependently reduces cholesterol uptake in Caco‐2 cells 42, suggesting that next to macrophage ChE the compound affects also other cell types and processes that can explain its overall beneficial effects on blood lipids. Therefore, particularly considering the effectiveness obtained in in vivo models, and especially those related to RCT 20, 21, 22, 23, 24, 42, piperine still remains an interesting bioactive natural product, and its mechanisms of action in this context remains highly relevant. Furthermore, characterization of molecular regulatory mechanisms able to influence ChE is of fundamental pathophysiological relevance in general.

Three transporter proteins located on the membrane of macrophages are crucial for cholesterol export, namely SR‐B1, ABCG1, and ABCA1 5. SR‐B1 is a bidirectional transmembrane transporter protein, contributing to the overall RCT by effects on both FC uptake and efflux 43. It is reported that SR‐B1 is widely expressed in liver cells (e.g., hepatocytes), leading to significant cholesterol uptake in this cell type 44, 45, 46, 47, 48. ABCG1 is an important transporter for macrophage ChE and it directs the effluxed intracellular FC for binding to HDL 49. The HDL‐associated cholesterol finally arrives at liver cells for uptake and further excretion from the body in the form of bile salts. It is a widely held view that ABCA1 is the most important cholesterol transporter protein for ChE in macrophages, mediating a large part of macrophage ChE 5. The FC transported through ABCA1 preferentially binds to apo A1 for further excretion via the RCT process 1. ABCA1 protein expression is regulated by the ABCA1 mRNA that are controlled by the activity of different transcription factors (e.g., nuclear receptors such as PPARs and LXRs) and epigenetic mechanisms (e.g., by miRNAs) 50, 51, 52. The upregulation of ABCA1 protein can be a result of PPARγ and LXRα activation associated with enhanced transcription of the ABCA1 gene 7, 8. Our study indicates that piperine induces ChE and upregulates ABCA1 protein levels. Though piperine could act as a PPARα agonist 53, piperine did not enhance mRNA levels of ABCA1 in our experiments, which is in line with former findings indicating that piperine might even antagonize the important transcriptional ABCA1 inducers PPARγ and LXRα in vivo 54, 55. Importantly, apo A1 was reported to stabilize ABCA1 protein by direct binding 56. On this background, it should be considered that in general piperine might also be able to act directly on lipoproteins, improving for example aspects related to HDL functional properties as cholesterol acceptor. Such putative improvements could be another possible mechanism responsible for an increase in ChE. One limitation of our work is that it does not explore this possibility. In the apo A1‐mediated ChE assay that we performed (Fig. 2A) cells are exposed to apo A1 for 6 h, followed by a 24 h piperine treatment. Nevertheless, the ABCA1‐stabilizing effect of piperine does not appear to be dependent on apo A1 treatment, because all other experiments in this study were performed without apo A1 exposure. In addition, the effect of piperine was evident already before the application of apo A1 (Fig. 4A), and piperine increased ChE to a similar extend as pioglitazone also when 1% human plasma was used as an acceptor instead of apo A1 (Fig. 2B). Therefore, considering the inability of piperine to induce ABCA1 mRNA level, we have further focused on studying ABCA1 posttranslational regulation, which is another important determinant of ABCA1 protein levels 9, 10, 11, 12, 13.

In the present study, we have analyzed the time‐dependent expression of ABCA1 protein, and found that piperine had its most potent effect 24 h after treatment (Fig. 4A). Therefore, the ABCA1 protein degradation rate was studied 24 h after piperine exposure. CHX, an inhibitor of de novo protein synthesis, was used to block further synthesis of ABCA1 in THP‐1 cells 13. In the absence of piperine, ABCA1 protein was degraded very fast, with only around 50% protein left 40 min after the application of CHX. However, in the presence of piperine, significantly higher (around 80%) ABCA1 levels were still present after 40 min of treatment with CHX. This result demonstrates an ABCA1 protein‐stabilizing effect of piperine.

So far, several previous studies have been investigating ABCA1 protein stability. It is known that a PEST sequence in the ABCA1 protein can be recognized by calpain, leading to the degradation of the protein 11. Based on the previous reports, next to calpain degradation two more important pathways were involved in ABCA1 degradation and ChE regulation in different cell types, in particular the proteasome‐ and lysosome‐mediated protein degradation pathways 9, 10, 11, 12. Notably, both lysosome‐ and calpain‐mediated degradation pathways are reported to be involved in ABCA1 degradation in THP‐1 cells in particular, which is also consistent with our findings (Fig. 5; 9, 10). Comparing the ABCA1 abundance for each condition after the application of pathway‐specific inhibitors (Fig. 5), we found that piperine mimics the effect of the calpain inhibitor, although piperine displayed a tendency to upregulate ABCA1 more than calpeptin alone, opening the possibility for existence of additional mechanisms upregulating ABCA1 protein. The mechanism by which piperine inhibits calpain activity remains unclear, although we are able to exclude a direct inhibition of calpain enzyme activity (Fig. 6), as well as changes in the protein levels of calpain 1 and calpain 2 (Supporting Information Fig. 2). Furthermore, siRNA knockdown of CAPNS1, a small heterodimer subunit essential for the function of both calpain 1 and calpain 2, did not affect the ABCA1‐stabilizing effect of piperine (Supporting Information Fig. 3). However, the outcome of the knockdown experiment should be interpreted with caution, because both siRNAs, scrambled siRNA and CAPNS1 siRNA, appear to affect basal expression levels of ABCA1 (Supporting Information Fig. 3). A calpain activity assay showed that, Z‐LLY‐FMK, an irreversible calpain inhibitor, significantly decreased calpain activity in THP‐1 macrophages. Though piperine also induced a significant reduction of calpain activity, the extent of the inhibition was not as strong as that of Z‐LLY‐FMK, and therefore possibly can not completely explain the entire observed ABCA1 upregulation. To unambiguously corroborate inhibition of calpain as mode of piperine's action, further siRNA or protein overexpression experiments need to be performed in future studies. In addition, calpain activity should be measured by a second method using a different principle (e.g., studying the cleavage of diverse endogenous calpain substrates), next to the fluorescent substrate‐conversion enzyme assay performed in this work (Fig. 6). Considering the magnitude of calpain inhibition by piperine, and that the ABCA1 gene transcription is not changed upon treatment with this compound, an additional posttranslational mechanism of piperine action cannot be excluded, which also deserves to be further addressed in future studies.

In conclusion, we show for the first time that piperine promotes ChE in THP‐1‐derived macrophages and upregulates ABCA1 protein level, which is mediated at least in part by inhibition of calpain activity.

*The authors have declared no conflict of interest*.

## Supporting information

Figure S1. ABCA1 protein expression in the absence and presence of piperine. Differentiated THP‐1‐derived macrophages were treated with solvent vehicle control (DMSO), or piperine (PIP; 50 μM). After 24 h incubation, the cells were lysed and 20 μg protein was resolved via SDS‐PAGE. Immunodetection was performed with antibody against ABCA1.
