# Supplementary material for: Piperine inhibits ABCA1 degradation and promotes cholesterol efflux from THP‐1‐derived macrophages
Source: Mol Nutr Food Res. 2016 Dec 23;61(4):1500960. doi: 10.1002/mnfr.201500960 (PMC5382977; doi:10.1002/mnfr.201500960)
Supplement: Supplementary file 1 — Figure S1. ABCA1 protein expression in the absence and presence of piperine. Differentiated THP‐1‐derived macrophages were treated with solvent vehicle control (DMSO), or piperine (PIP; 50 μM). After 24 h incubation, the cells were lysed and 20 μg protein was resolved via SDS‐PAGE. Immunodetection was performed with antibody against ABCA1. [file MNFR-61-na-s001.docx]

**Piperine Inhibits ABCA1 Degradation and Promotes Cholesterol Efflux from THP-1-derived Macrophages**

Limei Wang^1^, Veronika Palme^1^, Susanne Rotter^1^, Nicole Schilcher^1^, Malsor Cukaj^1^, Dongdong Wang^1^, Angela Ladurner^1^, Elke H. Heiss^1^, Herbert Stangl^2^, Verena M. Dirsch^1^, Atanas G. Atanasov^1,3,*^

*^1^Department of Pharmacognosy, University of Vienna, Vienna, Austria*

*^2^Institute of Medical Chemistry, Center for Pathobiochemistry and Genetics, Medical University of Vienna, Vienna, Austria*

*^3^Institute of Genetics and Animal Breeding of the Polish Academy of Sciences, 05-552 Jastrzebiec, Poland.*

**Keywords:** ABCA1, Calpain, Cholesterol efflux, Macrophages, Piperine

**Correspondence:** Dr. Atanas G. Atanasov, Department of Pharmacognosy, Faculty of Life Sciences, University of Vienna, Althanstrasse 14, A-1090 Vienna, Austria

**E-mail:** [atanas.atanasov@univie.ac.at](mailto:atanas.atanasov@univie.ac.at)

**Figure S1. ABCA1 protein expression in the absence and presence of piperine.** Differentiated THP-1-derived macrophages were treated with solvent vehicle control (DMSO), or piperine (PIP; 50 μM). After 24 h incubation, the cells were lysed and 20 μg protein was resolved via SDS-PAGE. Immunodetection was performed with antibody against ABCA1.

**Figure S2. Effect of piperine on calpain 1 and calpain 2 protein expression.** Differentiated THP-1-derived macrophages were treated with solvent vehicle control (DMSO) or piperine (PIP; 50 μM) for 24 h. After incubation, cells were lysed and 20 μg protein was resolved via SDS-PAGE. Immunodetection was performed with antibody against calpain 1 **(A)** and calpain 2 **(B)**, respectively. All data are mean ± S.D. (n=3) vs. solvent vehicle control (DMSO), n.s. no significance (paired *t*-tests).


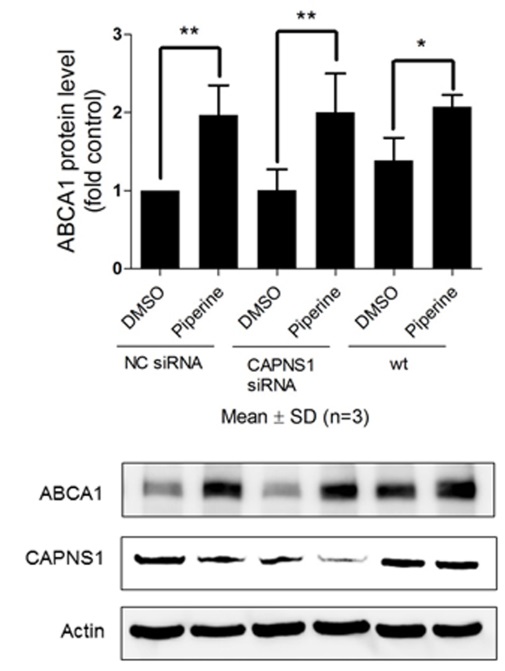


**Figure S3. Effect of siRNA knockdown of calpain small subunit 1 (CAPNS1) on ABCA1 protein expression.** Differentiated THP-1-derived macrophages were treated with scrambled siRNA (NC siRNA) or siRNA targeting calpain small subunit 1 (CAPNS1), and after 24 h also exposed to solvent vehicle control (DMSO) or piperine (100 μM) for another 24 h. After incubation, cells were lysed and 20 μg protein was resolved via SDS-PAGE. Immunodetection was performed with antibody against ABCA1, CAPNS1, and actin. Representative blots out of three performed experiments are presented. The data in the bar graph are presented as mean ± S.D. (n=3), **p <0.05, **p <0.01,* (paired *t*-tests).
